# Prenatal homelessness, food insecurity, and unemployment and adverse infant outcomes in a California cohort, 2007–2020

**DOI:** 10.1038/s41372-024-02161-5

**Published:** 2024-11-15

**Authors:** Lucia Ferrer, Christina Chambers, Anup Katheria, Annie Nguyen, Gretchen Bandoli

**Affiliations:** 1 Herbert Wertheim School of Public Health and Human Longevity Science, University of California San Diego, San Diego, CA USA; 2 Neonatal Research Institute, Sharp Mary Birch Hospital for Women and Newborns, San Diego, CA USA; 3 Department of Pediatrics, University of California San Diego, San Diego, CA USA

**Keywords:** Epidemiology, Outcomes research, Epidemiology, Population screening, Paediatrics

## Abstract

**Objectives:**

Characterize the relationship between infant outcomes and prenatal homelessness, food insecurity and unemployment.

**Study design:**

California live births between 22- and 44-weeks’ gestation comprised 6,089,327 pregnancies (2007–2020). Data were collected from linked Vital Statistics and hospital discharge records. Prenatal homelessness, food insecurity, and unemployment were classified as health-related social needs (HRSN) using International Classification of Disease codes in delivery records. Risk ratios for preterm birth, low birthweight, small for gestational age, neonatal intensive care unit admission, emergency department admission, rehospitalization, and death were estimated using log-linear Poisson regression adjusted for birthing person race, payer, and education.

**Results:**

65.7 per 100,000 births had HRSN. These infants had a higher risk of preterm birth (aRR 2.7), low birthweight (aRR 2.7), SGA (aRR 1.5), NICU admission (aRR 3.5), and death (aRR 3.0).

**Conclusions:**

HRSN increase the risk of infant morbidity and mortality but remain underreported in administrative records, making definitive conclusions difficult.

## Introduction

The US infant mortality rate (IMR) increased 3% from 2021 to 2022 (5.44 to 5.60 per 1000 live births), the first rise in over 20 years [1], and a rate far higher than in any other high-income country [2]. Concurrently, national rates of homelessness and economic instability are increasing rapidly. In California, over 171 000 individuals experience homelessness daily, 8–26% of whom are estimated to be pregnant [3]. Statewide surveys also suggest that 23% of all households and 28% of households with children experience food insecurity [4].

Social determinants of health (SDOH) likely contribute to the rising IMR and other adverse infant health outcomes. SDOH, which account for 30–55% of all health outcomes, are non-medical factors that influence health and well-being [5]. *Social risk factors (SRF)* refer to the individual-level SDOH that put an individual at risk for poor health [6]. Similarly, the term *health-related social needs (HRSN)* is used to refer to SRFs identified by a patient as an area of concern. While SRFs captured on screening tools are not always reflective of patients’ priorities or needs [6–8], HRSN, identified by patients themselves, may represent risk factors more accurate and relevant to their health, but their contribution to health outcomes remain poorly understood.

Pregnancy and the postpartum period can increase vulnerability to homelessness due to associated social, economic, and health challenges [3], and previous studies have demonstrated associations between unhoused status during pregnancy and mental illness, adverse birth outcomes, and maternal mortality [9–13]. Other HRSN have also been associated with adverse pregnancy and neonatal outcomes, including food insecurity [14–17] and economic instability [18–20].

To date, HRSN experienced during pregnancy, such as homelessness, inadequate housing, food insecurity, and unemployment, have not been systematically studied with infant outcomes. Given the high IMR in the US, it is of pressing importance to understand the association between HRSNs and infant outcomes. Thus, we aimed to: (1) characterize the prevalence of HRSN temporally and geographically, and (2) to examine the association between maternal HRSN and adverse infant outcomes.

## Methods

### Study design and population

We conducted a retrospective study of live-born births in California between 2007 and 2020, the most complete and recent data available. Data were collected from the Study of Outcomes in Mothers and Infants (SOMI), an administrative birth cohort derived from a complete collection of California birth records. These data are maintained by the California Vital Statistics and were linked to California Department of Health Care Access and Information (HCAI) hospital, emergency department, and ambulatory surgery records for the pregnant person and infant. The HCAI records include diagnosis and procedure codes recorded using the International Classification of Diseases, 9th Revision, Clinical Modification (ICD-9-CM) and the International Classification of Diseases, 10th Revision, Clinical Modification (ICD-10-CM). Our sample was limited to pregnant person-infant dyads for whom linkage was possible and excluded infants with major congenital anomalies (Supplementary Fig. 1). The SOMI study was approved by the Committee for the Protection of Human Subjects within the Health and Human Services Agency of the State of California and the University of California San Diego Human Research Protections Program.

### Study measures

Prenatal HRSN data were obtained from administrative hospital discharge records and classified using a composite approach based on the presence of any of the following HRSNs: homelessness, inadequate housing, unemployment, and food insecurity (ICD-9: V60.0 lack of housing, V60.1 inadequate housing, V62.0 unemployment; ICD-10: Z59.0 homelessness, Z59.1 inadequate housing; Z59.4 lack of adequate food, Z56.0 unemployment, unspecified). Health records queried included those of the pregnant person during the birth hospitalization.

Infant outcomes included preterm birth (<37 weeks’ gestation), low birth weight (LBW, <2500 g), small for gestational age (SGA) [21], neonatal intensive care unit (NICU) admission, emergency department admission (within the first year of life), rehospitalization (within the first year of life), and infant mortality (death within the first year of life). Specific causes of infant mortality were disaggregated into leading causes across the entire sample and separately for births with HRSN. The two leading causes of death across both samples, sudden unexpected infant death (SUID) and ‘certain conditions originating in the perinatal period’ (ICD-10CM codes P00-P96), were modeled as separate outcomes as well. SUID is a diagnosis created by a combination of ICD codes contained across two chapters: R95 (sudden infant death syndrome), R99 (unknown cause), and W75 (accidental suffocation or strangulation in bed). All infant outcomes were treated as dichotomous variables.

Various maternal data were selected to characterize the population with and without HRSN. Demographic information included self-reported race and ethnicity, maternal age at delivery, payment source at delivery, maternal education, body mass index (BMI), maternal place of birth, Special Supplemental Nutrition Program for Women, Infants, and Children (WIC) participation, and county of residence. Additional covariates collected included smoking during pregnancy, alcohol use disorder, substance use disorder, cannabis use disorder, inadequate prenatal care, diabetes, hypertensive disorder, preeclampsia, infection during pregnancy, and severe maternal morbidity. The data source, original form of each variable, and ICD codes used to identify each variable are available in Supplemental Table 1.

### Statistical analyses

HRSN frequency was calculated as a proportion of all individuals in the sample and for each specific HRSN. Maternal characteristics and perinatal comorbidities were summarized by HRSN. Chi-square tests were performed to compare proportions for categorical variables, and crude risk ratios (RRs) were calculated using log-linear Poisson regression models. The Wald test for trend was used to assess temporal trend in HRSN documentation. Log-linear Poisson regression models were used to estimate crude and adjusted RRs for the incidence of infant outcomes. Importantly, the Poisson distribution assumes the mean and variance are equal to each other. All outcome data modeled using Poisson regression were confirmed to meet this aforementioned assumption. These models were then adjusted for pregnant person education, race, and insurance payer at delivery. These covariates were chosen given that they are causes of both HRSN and adverse infant outcomes, and thus confound the observed relationship between the two. Despite the high prevalence of maternal comorbidities (e.g., high BMI, hypertension) and poor health behaviors (e.g., inadequate prenatal care) in our sample, and particularly among those with HRSN, these covariates were not included as confounders in our models given that they are likely not causes of HRSN, and thus more appropriately treated as mediators and subsumed into the total effects estimate. Choropleth maps at the California county level were created using county of residence at birth and shapefiles from the US Census Bureau.

Maternal HRSNs are risk factors for preterm birth and SGA, which are known risk factors for infant mortality [22, 23]. Thus, traditional mediation analyses were conducted for infant mortality overall, as well as for deaths attributed to SUID and ‘certain conditions originating in the perinatal period’, in order to quantify the excess risk of each outcome attributable to preterm birth or SGA (SAS macro *mediation*) [24, 25]. All models had a log link and were adjusted for the same covariates as previous models. Exposure-mediator interaction was included in the mediation analysis if the interaction term was significant in the regression model.

Lastly, missing values for education, BMI, hypertension and prenatal care adequacy were more common among individuals with HRSN. Since administrative databases may omit or erroneously document important confounders [26], a quantitative bias analysis was performed to evaluate unmeasured or residual confounding (R package *episensr*). We calculated E-values for all outcome models to assess the strength of an unmeasured confounder necessary to nullify our observed exposure-outcome associations. All data were analyzed using R (R Core Team, 2020) and RStudio (Rstudio Team, 2020) or SAS (SAS Institute Inc., Cary, NC, USA).

## Results

### Sample characteristics

6 089 327 pregnant individuals and their infants were included in our sample. 4 002 pregnant individuals had any documented HRSN (65.7 per 100 000 live births) (Table 1). The majority of HRSNs were related to housing concerns, and only thirteen individuals had documentation of food insecurity. Of note, a food insecurity diagnosis was only introduced upon the release of ICD 10 in 2015, and thus only available for data collected in and after 2015.

**Table 1:** Number and prevalence of maternal admissions with a HRSN.

	n	Prevalence per 100 000 live births
No HRSN	6 085 325	
Any HRSN	4 002	65.7
Housing	3 690	60.6
Homelessness	3 500	57.5
Inadequate Housing	191	3.1
Economic	333	5.5
Food Insecurity	13	0.21
Employment Problem	321	5.3

Food insecurity was added as a diagnosis upon the release of ICD-10 and is thus only available for births occurring during or after 2015.

Demographics for individuals who gave birth during the study period are provided in Table 2. Individuals with HRSN were more likely to be of Non-Hispanic White (32.4% vs 26.5%), Black (18.4% vs 5.0%), American Indian/Alaska Native (1.4% vs 0.3%), or Hawaiian/Pacific Islander (1.0% vs 0.3%) race compared to individuals without HRSN. Public insurance payer was more common among individuals with HRSN (82.3% vs 47.3%). Educational attainment less than 12 years (30.0% vs 18.7%) or equal to 12 years (34.0% vs 24.7%) was more common among individuals with HRSN. Individuals with HRSN were more likely to have been born in the United States (85.3% vs 59.6%).

**Table 2:** Maternal demographics and characteristics by HRSN status.

	No HRSN (*N* = 6 085 325)	HRSN (*N* = 4 002)	*p* value
Race, n (%)			
Non-Hispanic White	1 613 014 (26.5)	1 297 (32.4)	<0.001
Hispanic	3 018 087 (49.6)	1 367 (34.2)	<0.001
Black	304 191 (5.0)	738 (18.4)	<0.001
Asian	843 584 (13.9)	122 (3.1)	<0.001
American Indian/Alaska Native	19 926 (0.3)	56 (1.4)	<0.001
Hawaiian/Pacific Islander	23 709 (0.3)	39 (1.0)	<0.001
Other Race	306 449 (5.0)	478 (11.9)	<0.001
Two or More Races	127 235 (2.1)	211 (5.3)	<0.001
Not Stated/Unknown	132 211 (2.2)	169 (4.2)	<0.001
Maternal Age at Delivery, n (%)			
<18	116 330 (1.9)	40 (1.0)	<0.001
18–34	4 698 336 (77.2)	3 173 (79.3)	0.003
>34	1 270 427 (20.9)	789 (19.7)	<0.001
Missing	232 (0.004)	0 (0)	
Payer, n (%)			
Private	2 928 734 (48.1)	428 (10.7)	<0.001
Public	2 879 039 (47.3)	3 294 (82.3)	<0.001
Other	277 235 (4.6)	280 (7.0)	<0.001
Missing (NAs)	317 (0.0005)	0 (0)	
Education, n (%)			
Less Than 12 Years	1 139 386 (18.7)	1 200 (30.0)	<0.001
12 Years	1 500 458 (24.7)	1 360 (34.0)	<0.001
Greater than 12 Years	3 185 574 (52.3)	1 026 (25.6)	<0.001
Missing (NA)	259 907 (4.3)	416 (10.4)	<0.001
Body Mass Index, n (%)			
Underweight	249 312 (4.1)	213 (5.3)	<0.001
Normal	2 731 058 (44.9)	1 473 (36.8)	<0.001
Overweight	1 513 134 (24.9)	879 (22.0)	0.219
Obese	1 300 854 (21.4)	925 (23.1)	<0.001
Missing (NA)	290 967 (4.8)	512 (12.8)	<0.001
Maternal Place of Birth, n (%)			
United States	3 629 505 (59.6)	3 415 (85.3)	<0.001
Mexico	1 206 195 (19.8)	239 (6.0)	<0.001
Other	1 249 625 (20.5)	348 (8.7)	<0.001
WIC Participation, n (%)	3 006 918 (49.4)	2 212 (55.3)	<0.001
Smoked During Pregnancy, n (%)	145 030 (2.38)	1 694 (42.3)	<0.001
Substance Use Disorder During Pregnancy, n (%)	102 765 (1.69)	2 094 (52.3)	<0.001
Cannabis Use Disorder During Pregnancy, n (%)	62 544 (1.03)	947 (23.7)	<0.001
Alcohol Use Disorder During Pregnancy, n (%)	16 172 (0.27)	298 (7.44)	<0.001
Inadequate Prenatal Care, n (%)	1 454 218 (23.9)	2 397 (59.9)	<0.001
Missing PNC	221 496 (3.68)	274 (6.85)	<0.001
Diabetes, n (%)	637 112 (10.5)	520 (13.0)	<0.001
Any Hypertension, n (%)	515 676 (8.47)	874 (21.8)	<0.001
Missing Hypertension	33 866 (0.55)	78 (1.95)	<0.001
Preeclampsia, n (%)	251 665 (4.13)	514 (12.8)	<0.001
Infection, n (%)	561 766 (9.2)	1 430 (35.7)	<0.001
Severe Maternal Morbidity, n (%)	102 475 (1.68)	322 (8.05)	<0.001

Data are expressed as count (percentage). Characteristics that are not noted to have missing data are complete to the best of our knowledge.

The prevalence of perinatal comorbidities was high among individuals with HRSN (Table 2). Individuals with HRSN were more likely to smoke during pregnancy, have a substance, cannabis, and alcohol use disorder, receive inadequate prenatal care, have diabetes, hypertension, preeclampsia, and infection, and experience severe maternal morbidity during childbirth.

### Temporal trends

**Fig. 1:**
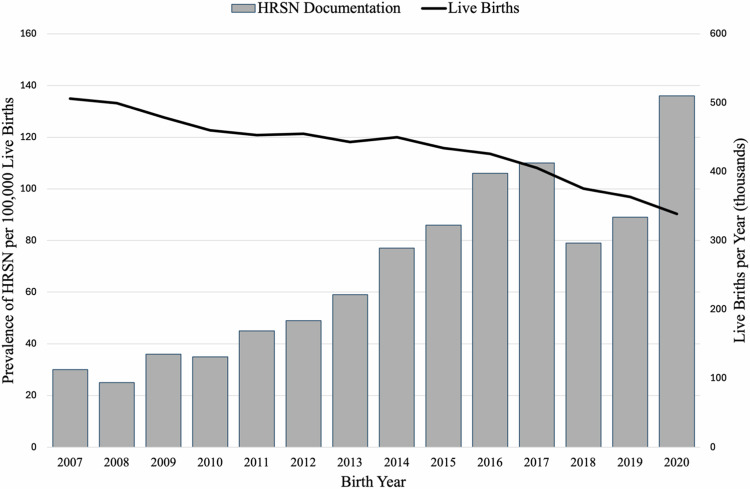
Prevalence of maternal HRSN among live births in California, 2007–2020. Food insecurity was added as a social code upon release of ICD 10, and is thus only available for births after 2015.

Figure 1 shows the prevalence of maternal HRSN and the live-birth rate across the study period. HRSN increased by an average of 12% per year across the study period (β = 0.12, *p* < 0.001).

### Geographic trends

**Fig. 2:**
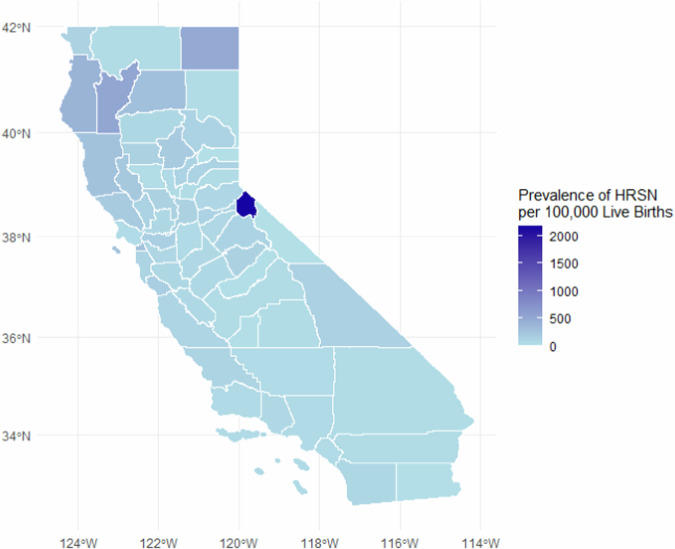
Map of maternal HRSNs among residents of California counties.

Figure 2 presents the prevalence of HRSN per 100 000 live births in each California county. HRSN prevalence was relatively homogenous across counties in central and southern California. Births in Alpine County had the highest prevalence of HRSN (2 170 per 100 000). HRSN was also more prevalent among residents of northern California counties, including Trinity (510 per 100 000), Modoc (470 per 100 000), and Humboldt (380 per 100 000) counties. Residents of Mono and Sierra counties reported no HRSN.

### Infant outcomes

Prevalence estimates for preterm birth (8.0% vs 23.7%), LBW (6.1% vs 18.1%), and NICU admission (4.9% vs 20%) were significantly higher among individuals with HRSN. Prevalence estimates for infant mortality were over four times higher among infants prenatally exposed to HRSN, though absolute prevalence remained low (0.3% vs 1.3%). However, a lesser proportion of infants born to individuals with HRSN were admitted to the emergency department in the first year of life, and there was no difference in rehospitalization between infants born to individuals with or without HRSN (Table 3).

**Table 3:** Infant outcomes by maternal HRSN status.

	No HRSN (*N* = 6 085 325)	HRSN (*N* = 4 002)	Unadjusted risk ratio (95% CI)	Adjusted^a^ risk ratio (95% CI)	E-value (Lower CI)
Preterm birth	484 123 (8.0)	947 (23.7)	3.0 (2.8, 3.2)	2.7 (2.5, 2.9)	4.8 (4.4)
Low birth weight	372 246 (6.1)	724 (18.1)	3.0 (2.75, 3.2)	2.7 (2.5, 2.9)	4.8 (4.4)
Small for gestational age	561 066 (9.2)	610 (15.2)	1.7 (1.53, 1.80)	1.5 (1.4, 1.6)	2.4 (2.1)
NICU admission	295 612 (4.9)	809 (20.2)	4.2 (3.9, 4.5)	3.5 (3.3, 3.8)	6.5 (6.0)
Infant ED admission	1 746 601 (28.7)	923 (23.1)	0.80 (0.8, 0.9)	0.7 (0.6, 0.7)	2.4 (2.12)
Infant readmission	563 121 (9.3)	353 (8.8)	1.0 (0.9, 1.1)	0.9 (0.8, 1.0)	1.5 (1.2)
Infant death	18 576 (0.3)	51 (1.3)	4.2 (3.1, 5.4)	3.0 (2.2, 4.1)	5.5 (3.9)
SUID^b^	2 606 (0.04)	11 (0.3)	6.4 (3.3, 11.0)	3.4 (1.9, 6.1)	6.2 (3.1)
P00-P96^c^	7 568 (0.1)	22 (0.6)	4.4 (2.8, 6.5)	3.9 (2.5, 5.9)	7.2 (4.5)

Data are expressed as count (percentage).

*NICU* neonatal intensive care unit, *ED* emergency department.

^a^Models adjusted for maternal education, race, and insurance payer at delivery.

^b^R95 (sudden infant death syndrome), R99 (unknown cause), and W75 (accidental suffocation or strangulation in bed) make up the diagnosis of sudden unexpected infant death.

^c^Certain conditions originating in the perinatal period (ICD-10CM codes P00-P96).

After adjustment for confounding variables (pregnant person education, race, and insurance payer at delivery), infants with maternal HRSN had a higher risk of preterm birth (aRR = 2.2, 95% CI 2.9, 3.5), LBW (aRR = 2.7, 95% CI 2.5, 2.9), SGA (aRR = 1.5, 95% CI 1.4, 1.6), NICU admission (aRR = 3.5, 95% CI 3.3, 3.8), and all-cause mortality (aRR = 3.0, 95% CI 2.2, 4.1) compared to infants without maternal HRSN. However, infants with maternal HRSN were less likely be admitted to the emergency department in the first year of life (aRR = 0.7, 95% CI 0.6, 0.7), and the risk of rehospitalization was similar between both groups (aRR = 0.9, 95% CI 0.8, 1.0). Infant mortality attributable to SUID and certain conditions originating in the perinatal period (ICD P00-P96) were also modeled in log-linear Poisson regression analyses as they were the two leading causes of death in the sample. Infants with maternal HRSN had a higher risk of SUID (aRR = 3.4, 95% CI 1.9, 6.1) and ‘certain conditions originating in the perinatal period’ (aRR = 3.9, 95% CI 2.5, 5.9) after adjustment for pregnant person race, education, and insurance payer (Table 3).

Results of the bias analysis (E-values and lower confidence intervals) are reported in Table 3. These E-values suggest that our models, except for infant rehospitalization, are robust and likely resistant to unmeasured confounding and missing data. To fully attenuate the RR for all-cause infant death, an unmeasured confounder would need to have a RR of 5.5 (lower CI of 3.9) with both the exposure (HRSN) and the outcome (all-cause infant death).

Mediation analyses were performed for infant deaths of all causes, deaths due to SUID, and deaths due to ‘certain conditions originating in the perinatal period’, as each were associated with maternal HRSN. 40% and 57% of the excess risk of all-cause infant death and death due to ‘certain conditions originating in the perinatal period’ was mediated by prematurity, respectively. Only 19% of the excess risk of death due to SUID was mediated by prematurity. SGA was not a strong mediator of infant death due to ‘certain conditions originating in the perinatal period’ (4%), SUID (5%), or infant death from any cause (5%) (Supplemental Table 2).

## Discussion

### Summary of findings

Using a large administrative cohort of over six million pregnant individuals and their infants, we found that maternal HRSN increased the risk of several adverse infant outcomes, including preterm birth, LBW, SGA, NICU admission, and infant death.

When disaggregated into specific causes of death, observed estimates of mortality appeared attributable to SUID and ‘conditions originating in the perinatal period.’ These findings suggest that though a significant proportion of deaths among infants prenatally exposed to HRSN occur at or shortly after delivery, infants were also at risk for death after discharge and through the first year of life.

Maternal HRSN was protective against infant emergency department admission and infant rehospitalization. Previous literature has demonstrated a positive relation between increased number of SRFs and infant emergency department admission and infant hospitalization [27, 28] using patient-endorsed social needs collected by survey. We speculate that the protective association observed in our study may be explained by the effect of prolonged NICU hospitalizations among preterm births, early death, or loss of custody by the birthing parent with HRSN, potentially resulting in a less hazardous environment for the infant. Additionally, this protective association may be explained by various social determinants of health that make healthcare access difficult, including un- or underinsurance, economic hardship, poor health literacy and transportation insecurity. Particularly, un- and underinsurance and economic hardship are some of the strongest predictors of delayed or forgone necessary medical care [29, 30], which may have influenced our observed relationship between HRSN, emergency department admission, and rehospitalization.

Previous literature has demonstrated similar results to our study. Green et al. reported similar increases in homelessness in a national sample of pregnant patients who had comparable rates of preterm birth and perinatal characteristics [10]. Similarly, Pantell et al. reported similar rates of HRSN and perinatal complications among pregnant individuals with HRSNs in a California sample [12]. It is possible that various unmeasured factors, downstream to HRSN, play a role in the observed maternal and infant morbidity and mortality associated with HRSNs. These include decreased access to primary and prenatal care services [31–33], lack of social support [9], increased rates of medical comorbidities [33, 34], and systemic bias and discrimination from healthcare systems and providers [35]. Further, other literature has found that unstable housing and poor infant health may be cyclical and co-constitutive, suggesting that these positive feedback loops can irreparably cause both poor health and lack of social opportunity for low-income families [36]. Given the high rates of maternal morbidity in our sample, it is difficult to ascertain the temporality of HRSN and poor health outcomes in our population. Birthing people in this sample may have experienced poor health outcomes leading to economic strain and subsequent housing instability or unemployment, or vice versa. Previous research suggests that a bidirectional relationship is most likely [11, 37, 38].

Though HRSN documentation increased over the study period, this increase likely reflects increasing ICD social code use rather than increasing prevalence of HRSN, and current rates of documentation likely still severely underestimate the true prevalence of HRSNs. This is probably due to a lack of validated SRF screening tools, limited and vague guidelines for SRF screening, and varying methods for documentation [7, 39]. In the future, standardized application of validated screening tools in clinical settings would allow providers to identify HRSN in a timely fashion and provide the foundation for research necessary to inform widespread SRF screening guidelines. Our findings bolster arguments for increased presence of ancillary staff and social workers in healthcare settings, whose presence improves health and utilization outcomes [40]. Additionally, HRSN data have the potential to affect clinical decision-making and policy strategies, and influence the design of interventions to address them.

Currently, use of ICD social codes is voluntary, and Centers for Medicare and Medicaid Services (CMS) and commercial payers offer no independent financial incentive for their use. Though these codes are not billable on their own, as of January 2021, CMS guidelines allow HRSNs to warrant a higher level of complexity for an office visit. This allows providers to bill for a moderate level of complexity—rather than a minimal or low level—given a HRSN that complicates the diagnosis or management of their patient’s condition [41].

### Limitations and future directions

First, this study is limited by its reliance on ICD codes for HRSN documentation. Despite our robust sample size, the prevalence of HRSN documentation likely represents a tremendous underreporting of SRF. We recognize that this limits our ability to make conclusions about the risks associated with these exposures, but this bias is likely to be true in any large epidemiological sample of similar data. Regardless, characterizing the prevalence of HRSN documentation is one of the primary aims of this paper, as we believe this information is important to inform future screening and documentation practices. Given the scarcity of SDOH screening and documentation guidelines in and out of pregnancy, different providers, health systems, and electronic medical records likely employ varied processes to document HRSN. Two recent studies estimated sensitivity of ICD social codes to be as low at 10% compared to patient screening responses [39, 42]. Thus, given the high rates of maternal morbidity among individuals with HRSN, HRSN documentation may be biased toward individuals with comorbid diagnoses (e.g., substance use disorders, severe maternal morbidity) or other risk factors (e.g., public insurance, inadequate prenatal care), which likely have independent negative effects on infant outcomes. This discrepancy would overestimate the effects of the relationship between HRSN and infant outcomes. However, given the low prevalence of HRSN in this sample, it is likely that a significant number of individuals experiencing SRFs but without a documented HRSN are included in our referent population, which likely attenuates our observed effect estimates. Due to these competing biases, certainty regarding the causal relationship between HRSN and adverse infant outcomes is limited, and likely vary by provider screening and documentation protocols. Further, the misclassification as compared to self-reported surveys may have affected the protective association we observed among infant emergency department admission and infant rehospitalization. Of note, 99.8% of individuals in our sample delivered in medical centers that had previously used ICD social codes, indicating that HRSN documentation is likely not differential by hospital.

Second, given the low prevalence of ICD social code documentation in this study, we were unable to disaggregate the effects of specific SRFs on infant outcomes. Food insecurity, unemployment, and homelessness during pregnancy likely confer varying amounts of risk to infant outcomes, and experiencing two or three risk factors simultaneously may be riskier than experiencing one. Given the high proportion of homelessness among individuals in our HRSN sample, it is likely that much of the risk of adverse infant outcomes is attributable to homeless status during pregnancy. Further, only thirteen individuals in our sample had a diagnosis of food insecurity, which constitutes a tremendous underreporting compared to reliable estimates that point to nearly 30% of families with children experiencing food insecurity [4]. The “Hunger Vital Sign”, a two-item food insecurity screening tool, is widely used in clinical practice given its ease of administration and validity; an affirmative response to either question one or two has a sensitivity of 97% and a specificity of 83%. Thus, the underreporting of food insecurity in our sample is likely the result of inadequate documentation rather than inadequate screening. Given that food insecurity and homelessness frequently exist in tandem [43], it is likely that many individuals with documentation of homelessness were also experiencing food insecurity.

Third, we did not have information on postnatal exposure to SRFs. Especially with outcomes like SUID, inadequate housing may increase the prevalence of bedsharing, unsafe sleeping environments, and secondhand marijuana or tobacco smoke, which are all risk factors for SUID and cannot be accounted for in this study [44]. This limited our ability to make conclusions about the effect of maternal HRSN versus infant HRSN on infant outcomes. Conversely, given the extensive housing and economic resources available to pregnant people and families in California [45], it is possible that many infants with prenatal HRSN experienced more favorable postnatal environments, which would serve as a protective factor against poor health outcomes in infancy and beyond.

Despite these limitations, a strength of this study is the use of a population-based administrative cohort of California births. The study population was diverse with respect to race and ethnicity, socioeconomic status, and geographic region. The large size of the sample allowed for robust estimations of the temporal trends in ICD social code use in the state, an important first step in increasing SRF screening and documentation during pregnancy.

## Conclusion

HRSN are underreported in administrative health records, and prenatal exposure to HRSN is a risk factor for adverse infant health outcomes and infant mortality. Our findings add to the growing body of literature characterizing the link between SRFs and health outcomes and reinforce the importance of screening and documentation of HRSNs. The risks associated with HRSN exposure should, at a minimum, prompt their use as a screening tool to determine those who would benefit from additional services. This information is vital to determine the causal pathways between SRFs and perinatal outcomes, and for developing effective interventions that improve the social environments, well-being, and health outcomes for those with HRSNs.

## Supplementary information

Supplemental Material

## Data Availability

The data that support the findings of this study are available from the California Department of Public Health (CDPH). Restrictions apply to the availability of these data, which were used under license for this study. Authors do not have permission to share data. We direct researchers to the CDPH Center for Health Statistics and Information, and the California Department of Health Care Access and Information for information on requesting and accessing California state data.

## References

[1] Danielle M Ely, Anne K Driscoll. Infant Mortality in the United States: Provisional Data From the 2022 Period Linked Birth/Infant Death File. *National Center for Health Statistics*; 2023. 10.15620/cdc:133699. 39680705

[2] Munira Z Gunja, Evan D Gumas, Reginald D. Williams IIUS Health Care from a Global Perspective, 2022: *Accelerating Spending, Worsening Outcomes*. *Commonwealth Fund*. Published online January 31, 2023. 10.26099/8ejy-yc74

[3] Kushel M, Moore T. *Toward a New Understanding: The California Statewide Study of People Experiencing Homelessness*. UCSF Benioff Homelessness and Housing Initiative; 2023.

[4] US Census Bureau. *Household Pulse Survey: Food Sufficiency and Food Security*. 2020. Accessed May 1, 2024. https://www.census.gov/data/tables/2023/demo/hhp/hhp63.html

[5] World Health Organization. *Social determinants of health. Social Determinants of Health*. 2023. Accessed October 26, 2023. https://www.who.int/health-topics/social-determinants-of-health

[6] Alderwick H, Gottlieb LM. Meanings and misunderstandings: a social determinants of health lexicon for health care systems. Milbank Q. 2019;97:407–19. 10.1111/1468-0009.12390 31069864 10.1111/1468-0009.12390 PMC6554506

[7] Eder M, Henninger M, Durbin S, Iacocca MO, Martin A, Gottlieb LM, et al. Screening and interventions for social risk factors: technical brief to support the US preventive services task force. JAMA. 2021;326:1416–28. 10.1001/jama.2021.12825 34468710 10.1001/jama.2021.12825 PMC13460784

[8] Green K, Zook M. When Talking About Social Determinants, Precision Matters. *Health Affairs Forefront*. 10.1377/forefront.20191025.776011

[9] American College of Obstetricians and Gynecologists. *Health Care for Homeless Women*. 2023. Accessed December 28, 2023. https://www.acog.org/clinical/clinical-guidance/committee-opinion/articles/2013/10/health-care-for-homeless-women

[10] Green JM, Fabricant SP, Duval CJ, Panchal VR, Cahoon SS, Mandelbaum RS, et al. Trends, characteristics, and maternal morbidity associated with unhoused status in pregnancy. JAMA Netw Open. 2023;6:e2326352. 10.1001/jamanetworkopen.2023.26352 37523185 10.1001/jamanetworkopen.2023.26352 PMC10391303

[11] Leifheit KM, Schwartz GL, Pollack CE, Edin KJ, Black MM, Jennings JM, et al. Severe housing insecurity during pregnancy: association with adverse birth and infant outcomes. Int J Environ Res Public Health. 2020;17:8659. 10.3390/ijerph17228659 33233450 10.3390/ijerph17228659 PMC7700461

[12] Pantell MS, Baer RJ, Torres JM, Felder JN, Gomez AM, Chambers BD, et al. Associations between unstable housing, obstetric outcomes, and perinatal health care utilization. Am J Obstet Gynecol MFM. 2019;1:100053. 10.1016/j.ajogmf.2019.100053 33345843 10.1016/j.ajogmf.2019.100053

[13] Huang K, Waken RJ, Luke AA, Carter EB, Lindley KJ, Joynt Maddox KE. Risk of delivery complications among pregnant people experiencing housing insecurity. Am J Obstet Gynecol MFM. 2023;5:100819. 10.1016/j.ajogmf.2022.100819 36436788 10.1016/j.ajogmf.2022.100819

[14] Pasha VC, Gerchow L, Lyndon A, Clark-Cutaia M, Wright F. Understanding food insecurity as a determinant of health in pregnancy within the United States: an integrative review. Health Equity. 2024;8:206–25. 10.1089/heq.2023.0116 38559844 10.1089/heq.2023.0116 PMC10979674

[15] Shreffler KM, Dressler CM, Ciciolla L, Wetherill MS, Croff JM. Maternal periconception food insecurity and postpartum parenting stress and bonding outcomes. Front Nutr. 2024;11:1275380. 10.3389/fnut.2024.1275380 38468697 10.3389/fnut.2024.1275380 PMC10925610

[16] Laraia BA, Gamba R, Saraiva C, Dove MS, Marchi K, Braveman P. Severe maternal hardships are associated with food insecurity among low-income/lower-income women during pregnancy: results from the 2012–2014 California maternal infant health assessment. BMC Pregnancy Childbirth. 2022;22:138. 10.1186/s12884-022-04464-x 35183141 10.1186/s12884-022-04464-x PMC8858559

[17] Augusto ALP, de Abreu Rodrigues AV, Domingos TB, Salles-Costa R. Household food insecurity associated with gestacional and neonatal outcomes: a systematic review. BMC Pregnancy Childbirth. 2020;20:229. 10.1186/s12884-020-02917-9 32303221 10.1186/s12884-020-02917-9 PMC7164154

[18] Pawar D, Sarker M, Caughey AB, Valent AM. Influence of socioeconomic status on adverse outcomes in pregnancy [15C]. Obstet Gynecol. 2020;135:33S. 10.1097/01.AOG.0000663292.11789.bf 10.1007/s10995-023-03701-9 37273137

[19] Bushnik T, Yang S, Kaufman JS, Kramer MS, Wilkins R. Socioeconomic disparities in small-for-gestational-age birth and preterm birth. Health Rep. 2017;28:3–10. 29140535

[20] Joseph KS, Liston RM, Dodds L, Dahlgren L, Allen AC. Socioeconomic status and perinatal outcomes in a setting with universal access to essential health care services. CMAJ. 2007;177:583–90. 10.1503/cmaj.061198 17846440 10.1503/cmaj.061198 PMC1963370

[21] Talge NM, Mudd LM, Sikorskii A, Basso O. United States birth weight reference corrected for implausible gestational age estimates. Pediatrics. 2014;133:844–53. 10.1542/peds.2013-3285 24777216 10.1542/peds.2013-3285

[22] Ray JG, Park AL, Fell DB. Mortality in infants affected by preterm birth and severe small-for-gestational age birth weight. Pediatrics. 2017;140:e20171881. 10.1542/peds.2017-1881 29117948 10.1542/peds.2017-1881

[23] World Health Organization. *Preterm birth*. May 10, 2023. Accessed January 17, 2024. https://www.who.int/news-room/fact-sheets/detail/preterm-birth

[24] Valeri L, VanderWeele TJ. Mediation analysis allowing for exposure-mediator interactions and causal interpretation: theoretical assumptions and implementation with SAS and SPSS macros. Psychol Methods. 2013;18:137–50. 10.1037/a0031034 23379553 10.1037/a0031034 PMC3659198

[25] Rijnhart JJM, Lamp SJ, Valente MJ, MacKinnon DP, Twisk JWR, Heymans MW. Mediation analysis methods used in observational research: a scoping review and recommendations. BMC Med Res Methodol. 2021;21:226. 10.1186/s12874-021-01426-3 34689754 10.1186/s12874-021-01426-3 PMC8543973

[26] Tawfik DS, Gould JB, Profit J. Perinatal risk factors and outcome coding in clinical and administrative databases. Pediatrics. 2019;143:e20181487. 10.1542/peds.2018-1487 30626622 10.1542/peds.2018-1487 PMC6361352

[27] Rigdon J, Montez K, Palakshappa D, Brown C, Downs SM, Albertini LW, et al. Social risk factors influence pediatric emergency department utilization and hospitalizations. J Pediatr. 2022;249:35–42.e4. 10.1016/j.jpeds.2022.06.004 35697140 10.1016/j.jpeds.2022.06.004 PMC11210599

[28] Wurster Ovalle VM, Beck AF, Ollberding NJ, Klein MD. Social risk screening in pediatric primary care anticipates acute care utilization. Pediatr Emerg Care. 2021;37:e609–e614. 10.1097/PEC.0000000000001979 32149994 10.1097/PEC.0000000000001979

[29] Wisk LE, Witt WP. Predictors of delayed or forgone needed health care for families with children. Pediatrics. 2012;130:1027–37. 10.1542/peds.2012-0668 23129081 10.1542/peds.2012-0668 PMC3507252

[30] Galbraith AA, Soumerai SB, Ross-Degnan D, Rosenthal MB, Gay C, Lieu TA. Delayed and forgone care for families with chronic conditions in high-deductible health plans. J Gen Intern Med. 2012;27:1105–11. 10.1007/s11606-011-1970-8 22249829 10.1007/s11606-011-1970-8 PMC3514993

[31] Richards R, Merrill RM, Baksh L. Health behaviors and infant health outcomes in homeless pregnant women in the United States. Pediatrics. 2011;128:438–46. 10.1542/peds.2010-3491 21824881 10.1542/peds.2010-3491

[32] DiTosto JD, Holder K, Soyemi E, Beestrum M, Yee LM. Housing instability and adverse perinatal outcomes: a systematic review. Am J Obstet Gynecol MFM. 2021;3:100477. 10.1016/j.ajogmf.2021.100477 34481998 10.1016/j.ajogmf.2021.100477 PMC9057001

[33] Clark RE, Weinreb L, Flahive JM, Seifert RW. Homelessness contributes to pregnancy complications. Health Aff. 2019;38:139–46. 10.1377/hlthaff.2018.05156 10.1377/hlthaff.2018.05156 30615521

[34] St Martin BS, Spiegel AM, Sie L, Leonard SA, Seidman D Girsen AI. et al. Homelessness in pregnancy: perinatal outcomes. J Perinatol. 2021;41:2742–8. 10.1038/s41372-021-01187-3 34404925 10.1038/s41372-021-01187-3 PMC9507167

[35] Allen J, Vottero B. Experiences of homeless women in accessing health care in community-based settings: a qualitative systematic review. JBI Evid Synth. 2020;18:1970. 10.11124/JBISRIR-D-19-00214 32813421 10.11124/JBISRIR-D-19-00214

[36] Schwartz GL, Leifheit KM, Berkman LF, Chen JT, Arcaya MC. Health selection into eviction: adverse birth outcomes and children’s risk of eviction through age 5 years. Am J Epidemiol. 2021;190:1260–9. 10.1093/aje/kwab007 33454765 10.1093/aje/kwab007 PMC8484772

[37] Himmelstein G, Desmond M. Association of eviction with adverse birth outcomes among women in Georgia, 2000 to 2016. JAMA Pediatr. 2021;175:494–500. 10.1001/jamapediatrics.2020.6550 33646291 10.1001/jamapediatrics.2020.6550 PMC7922232

[38] Khadka A, Fink G, Gromis A, McConnell M. In utero exposure to threat of evictions and preterm birth: Evidence from the United States. Health Serv Res. 2020;55:823–32. 10.1111/1475-6773.13551 32976630 10.1111/1475-6773.13551 PMC7518827

[39] Iott BE, Rivas S, Gottlieb LM, Adler-Milstein J, Pantell MS. Structured and unstructured social risk factor documentation in the electronic health record underestimates patients’ self-reported risks. J Am Med Inform Assoc. 2024;31:714–9. 10.1093/jamia/ocad261 38216127 10.1093/jamia/ocad261 PMC10873825

[40] Steketee G, Ross AM, Wachman MK. Health outcomes and costs of social work services: a systematic review. Am J Public Health. 2017;107:S256–S266. 10.2105/AJPH.2017.304004 29236534 10.2105/AJPH.2017.304004 PMC5731071

[41] American Medical Association. *CPT evaluation and management (E/M) office or other outpatient (99202−99215) and prolonged services (99354:99355, 99356, 99417) code and guideline changes. Published online January 1, 2021. Accessed* October 27, 2023. https://www.ama-assn.org/system/files/2019-06/cpt-office-prolonged-svs-code-changes.pdf.

[42] Vest JR, Wu W, Mendonca EA. Sensitivity and specificity of real-world social factor screening approaches. J Med Syst. 2021;45:111. 10.1007/s10916-021-01788-7 34767091 10.1007/s10916-021-01788-7 PMC8588755

[43] Lee CY, Zhao X, Reesor-Oyer L, Cepni AB, Hernandez DC. Bidirectional relationship between food insecurity and housing instability. J Acad Nutr Diet. 2021;121:84–91. 10.1016/j.jand.2020.08.081 33060025 10.1016/j.jand.2020.08.081

[44] National Institute of Child Health and Human Development. Safe to Sleep®: What Are the Known Risk Factors? Accessed April 15, 2024. https://safetosleep.nichd.nih.gov/about/risk-factors

[45] California Department of Social Services. Housing Programs. Accessed September 26, 2024. https://www.cdss.ca.gov/inforesources/cdss-programs/housing-programs

