# Supplementary material for: Prenatal homelessness, food insecurity, and unemployment and adverse infant outcomes in a California cohort, 2007–2020
Source: J Perinatol. 2024 Nov 15;45(11):1528–34. doi: 10.1038/s41372-024-02161-5 (PMC12660135; doi:10.1038/s41372-024-02161-5)
Supplement: Supplementary file 1 — Supplemental Material [file 41372_2024_2161_MOESM1_ESM.docx]

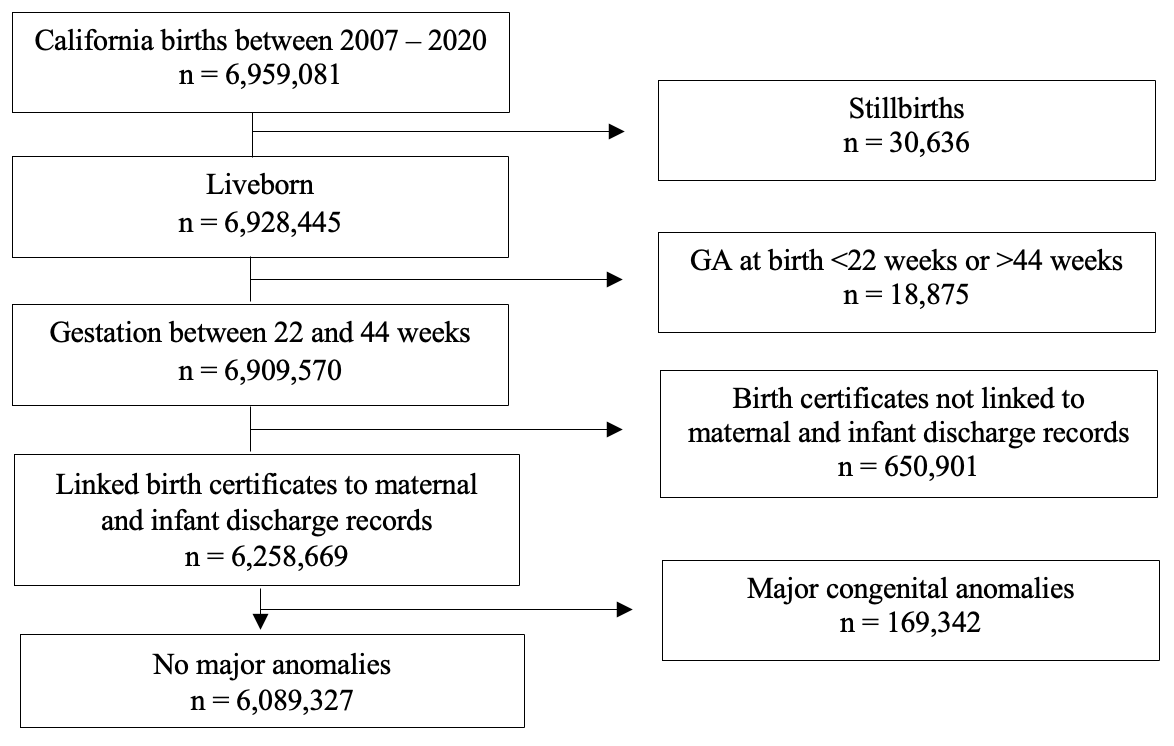


Supplemental Figure 1. Study Sample Selection

Supplemental Table 1. Data source, variable form, and relevant ICD codes for each variable

| Variable | Data Source | Notes/ICD Codes |
| --- | --- | --- |
| Health Related Social Need | HCAI | ICD-9: V60.0 lack of housing, V60.1 inadequate housing, V62.0 unemployment; ICD-10: Z59.0 homelessness, Z59.1 inadequate housing; Z59.4 lack of adequate food, Z56.0 unemployment, unspecified |
| Race/Ethnicity | Birth records | Hispanic, White non-Hispanic, Black non-Hispanic, Asian non-Hispanic, other race non-Hispanic, American Indian/Alaska Native non-Hispanic, Hawaiian/Pacific Islander non-Hispanic, two or more races non-Hispanic, unknown race non-Hispanic |
| Maternal Age at Delivery | Birth records | Continuous variable |
| Insurance Payer | Birth records | Private insurance, No payer, Medi-Cal, Other government insurance, Self-pay, Other payment, TriCare, Unknown payer |
| Maternal Education | Birth records | Did not attend, 1^st^-11^th^ grade, 12^th^ grade no diploma, HS graduate, GED, some college, Associate, Bachelor, Masters, Doctorate, Professional |
| Body Mass Index | Birth records | Calculated from height and pre-pregnancy weight |
| Maternal Place of Birth | Birth records | United States, Mexico, Other |
| WIC Participation | Birth records | Yes, No, Unknown/Not Stated |
| County of Residence | Birth records | CA county codes (001-058) |
| State of Residence | Birth records | US state codes (101-151) |
| Birth Year | Birth records | Continuous variable |
| Smoked During Pregnancy | HCAI, birth records | ICD-9 649.0 or ICD-10 O99.33  Coded if present in either |
| Substance Use Disorder | HCAI | ICD-9 304, 305.0. 305.2-305.9, 648.3, ICD-10 F11, F12, F13, F14, F15, F16, F18, F19 |
| Cannabis Use Disorder | HCAI | ICD-9 306.2, 304.3; ICD-10 F12 |
| Alcohol Use Disorder | HCAI | ICD-9 303, 305.0; ICD-10 O99.31, F10 |
| Preterm Birth | Birth records | Continuous variable |
| Prenatal Care | HCAI | Adequate plus, adequate, intermediate, and inadequate prenatal care variables created using recorded month of prenatal care initiation and number of prenatal care visits per Kotelchuck et al. |
| Preexisting diabetes | HCAI, birth records | ICD-9 648.0, 250; ICD-10 infant P70.1, maternal O24.0, O24.1, O24.2, O24.3, E10, E11, E12, E13, E14. Birth records: Pre-pregnancy diabetes (diagnosis prior to this pregnancy) |
| Gestational diabetes | HCAI, birth records | ICD-9 648.8; ICD-10 infant P70.0, maternal O24.4. Birth records: gestational diabetes (diagnosis in this pregnancy) |
| Hypertensive Disorder | HCAI | ICD-9 642; ICD-10 O1 |
| Preeclampsia | HCAI | ICD-9 642.4, 642.5, 642.6, 642.7; ICD-10 O11, O14.0, O14.1, O14.2, O14.9, O15. Birth records: preeclampsia |
| Infection | HCAI, birth records | Maternal codes: ICD-9 647, 646.5, 646.6; ICD-10 P90, O23.0, O23.1, O23.2, O23.3, O23.4. Infant codes: ICD-9 760.2, 760.1. Birth records: chlamydia, gonorrhea, syphilis, hepatitis C, hepatitis B (acute infection or carrier) |
| Severe Maternal Morbidity | HCAI, birth records | ICD 9 and ICD 10 codes corresponding to 21 indicators per CDC. |
| Low Birth Weight | Birth records | Continuous variable |
| Small for Gestational Age | Birth records | Calculated using infant sex, gestational age at delivery, and birthweight per Talge, et al, 2014 |
| Infant Emergency Department Admission | HCAI | Binary (yes, no) |
| Infant Rehospitalization | HCAI | Binary (yes, no) |
| Infant Death | Death records | ICD-9 and ICD-10 codes |
| County of Residence | Birth records | FIPS codes |

Supplemental Table 2. Mediation analysis for HRSN status and select cause of death.

|  | **Preterm Birth^a^** | | | **Small for Gestational Age^a^** | | |
| --- | --- | --- | --- | --- | --- | --- |
|  | **Natural direct effect** | **Natural indirect effect** | **Proportion mediated** | **Natural direct effect** | **Natural indirect effect** | **Proportion mediated** |
| Any cause of death | 2.3^+^ (1.6, 3.2) | 1.4 (1.2, 1.6) | 40% | 3.0 (2.2, 4.1) | 1.0 (1.0, 1.0) | 5% |
| Sudden unexpected infant death^b^ | 2.9 (1.6, 5.3) | 1.2 (1.1, 1.2) | 19% | 3.3 (1.8, 6.0) | 1.0 (1.0, 1.0) | 5% |
| Certain conditions originating in the perinatal period(P00-P96) | 2.4^+^ (1.4, 3.9) | 1.7 (1.3, 2.3) | 57% | 3.9 (2.6, 6.1) | 1.0 (1.0, 1.0) | 4% |
| ^a^ Models adjusted for maternal education, race, and insurance payer at delivery  ^b^ R95 (sudden infant death syndrome), R99 (unknown cause), and W75 (accidental suffocation or strangulation in bed) make up the diagnosis of sudden unexpected infant death  ^+^Exposure-mediator interaction included in mediation analysis | | | | | | |
